# P04 An evaluation of antimicrobial stewardship education in the School of Pharmacy of an Irish university—a mixed methods study

**DOI:** 10.1093/jacamr/dlac004.003

**Published:** 2022-02-16

**Authors:** T. M. Barbosa, A. Fleming, E. Crowley, A. O’Connor, L. Costello, S. McCarthy

**Affiliations:** Pharmaceutical Care Research Group, School of Pharmacy, University College Cork, Ireland; Pharmaceutical Care Research Group, School of Pharmacy, University College Cork, Ireland; Pharmacy Department, Mercy University Hospital, Cork, Ireland; Pharmaceutical Care Research Group, School of Pharmacy, University College Cork, Ireland; Pharmaceutical Care Research Group, School of Pharmacy, University College Cork, Ireland; Pharmaceutical Care Research Group, School of Pharmacy, University College Cork, Ireland; Pharmaceutical Care Research Group, School of Pharmacy, University College Cork, Ireland

## Abstract

**Background:**

Antimicrobial stewardship (AMS) is essential to control the emergence of antimicrobial resistance (AMR) which has become an international health priority. Education of undergraduate students on AMS and AMR is a strategic objective in the WHO Global Action Plan on AMR and Ireland's National Action Plan.^1,2^ Research on AMS/AMR education has focused primarily on medical students with less emphasis on those from other healthcare profession including pharmacy students.

**Objectives:**

To investigate AMS and AMR education in the Pharmacy undergraduate programme in the School of Pharmacy of an Irish University.

**Methods:**

A mixed methods study was conducted. Ten semi-structured interviews were conducted in October 2019 with academic staff to capture their views on AMS education. Participants included staff members from different disciplines, those identified as involved in teaching elements of infectious disease, antibiotics, AMS and related topics, and those involved in curriculum design and approval within the School of Pharmacy. Interview transcripts were analysed by thematic analysis. An electronic survey of 17 questions was emailed to all second to fifth year UCC Pharmacy students in October 2019 to gather students’ views and experiences of AMS education. The survey contained four sections which addressed demographics, AMS, resources and education using open-ended, closed-ended or Likert-scale questions. Ethical approval was obtained.

**Results:**

Six key themes were identified from the ten interviews: (i) curriculum priorities and capacity; (ii) housing of the subject, fragmentation and cohesion; (iii) integration; (iv) communication; (v) teaching methods; and (vi) assessment methods. 113 students participated in the survey (32.3% response rate). 96% agree that a strong knowledge of antimicrobials for their future careers is important, and over 89% of students desire more education on AMR and AMS. Only 43% of students found their AMS education provided sufficient preparation for practice. Students felt most prepared to recognize clinical signs of infection and least prepared for IV to oral switching, interpreting biological marker and de-escalation of antimicrobials. Over 50% of students never used or were not familiar with the national primary care antimicrobial guidelines: www.antibioticprescribing.ie.

**Conclusions:**

Pharmacy students and staff agree that AMS and AMR are important and need enhanced educational focus. Students feel there are gaps in their AMS education and expressed a desire for more education in this area. Improved communication and identification of curriculum priorities by staff could help to establish a more cohesive and comprehensive educational approach to this area.
